# Supplementary material for: Heat adaptation of phage T7 under an extended genetic code
Source: Virus Evol. 2021 Dec 1;7(2):veab100. doi: 10.1093/ve/veab100 (PMC8923235; doi:10.1093/ve/veab100)
Supplement: veab100_Supp [file veab100_supp.zip › Sequencing_Analysis.zip!/Sequencing_Analysis/S2/evidence/JC_SIDE_1_77.html]

BRESEQ :: Evidence


| New junction evidence | | | | | | | | | | | |
| --- | --- | --- | --- | --- | --- | --- | --- | --- | --- | --- | --- |
|  | | seq id | position | reads (cov) | reads (cov) | score | skew | freq | annotation | gene | product |
| \* | ? | NC\_001604.1 | = 36882 | 5203 (0.980) | 883 (0.170) | 243/582 | 5.8 | 15.5% | intergenic (–/–) | *–/–* | –/– |
|  | ? | NC\_001604.1 | 37265 = | 4556 (0.890) |  |  |  |  | intergenic (–/–) | *–/–* | –/– |
| Rejected: Coverage evenness skew score above cutoff. | | | | | | | | | | | |
| Rejected: Frequency below/above cutoff threshold. | | | | | | | | | | | |

Only 100 of 5314 total aligned reads displayed.  

`CTTAGAGATGTTGGACACTGCGATGGCTCAGCGTATGCTTGCGGACCTTTCGGACCATGAGCGTCGCTCTCCGCAACTCTATAATGCTATTAACAAACTGTTAGACCGCCACAAGTTCCAGATTGGTAAGTTGCAGCCGGATGTTCACATCTTAGGTGGCCTTGCTGGTGCTCTTGAAGAGTACAAAGAGAAAGTCGGTGATAACGGTCTTACGGATGATGATATTTACACATTACAGTGATATACTCAAGGCCACTACAGATAGTGGTCTTTATGGATGTCATTGTCTATACGAGATG‑CTCCTACGTGAAATCTGAAAGTTAACGGGAGGCATTATGCTAGAATTTTTACGTAAGCTAATCCCTTGGGTTCTCGCTGGGATGCTATTCGGGTTAGGATGGCATCTAGGGTCAGACTCAATGGACGCTAAATGGAAACAGGAGGTACACAATGAGTACGTTAAGAGAGTTGAGGCTGCGAAGAGCACTCAAAGAGCAATCGATGCGGTATCTGCTAAGTATCAAGAAGACCTTGCCGCGCTGGAAGGGAGCACTGATAGGATTATTTCTGATTTGCGTAGCGACAATAAGCGGTTGCGCGT  >  NC_001604.1/36582‑37182`  

                                                                                                                                                                                                                                                                                                             `|`                                                                                                                                                                                                                                                                                                              
 `ttAGAGATGTTGGACACTGCGATGGCTCAGCGTATGCTTGCGGACCTTTCGGACCATGAGCGTCGCTCTCCGCAACTCTATAATGCTATTAACAAACTGTTAGACCGCCACAAGTTCCAGATTGGTAAGTTGCAGCCGGATGTTCACATCTTAGGTGGCCTTGCTGGTGCTCTTGAAGAGTACAAAGAGAAAGTCGGTGATAACGGTCTTACGGATGATGATATTTACACATTACAGTGATATACTCAAGGCCACTACAGATAGTGGTCTTTATGGATGTCATTGTCTATACGAGATG‑CTc                                                                                                                                                                                                                                                                                                             <  1:387434/301‑1 (MQ=255)`  
  `tAGAGAGGTTGGACACTGCGATGGCTCAGCGTATGCTTGCGGACCTTTCGGACCATGAGCGTCGCTCTCCGCAACTCTATAATGCTATTAACAAACTGTTAGACCGCCACAAGTTCCAGATTGGTAAGTTGCAGCCGGATGTTCACATCTTAGGTGGCCTTGCTGGTGCTCTTGAAGAGTACAAAGAGAAAGTCGGTGATAACGGTCTTACGGATGATGATATTTACACATTACAGTGATATACTCAAGGCCACTACAGATAGTGGTCTTTATGGATGTCATTGTCTATACGAGATG‑CTcc                                                                                                                                                                                                                                                                                                            <  1:100252/301‑1 (MQ=255)`  
    `gagaTGTTGGACACTGCGATGGCTCAGCGTATGCTTGCGGACCTTTCGGACCATGAGCGTCGCTCTCCGCAACTCTATAATGCTATTAACAAACTGTTAGACCGCCACAAGTTCCAGATTGGTAAGTTGCAGCCGGATGTTCACATCTTAGGTGGCCTTGCTGGTGCTCTTGAAGAGTACAAAGAGAAAGTCGGTGATAACGGTCTTACGGATGATGATATTTACACATTACAGTGATATACTCAAGGCCACTACAGATAGTGGTCTTTATGGATGTCATTGTCTATACGAGATG‑CTCCTa                                                                                                                                                                                                                                                                                                          <  1:264154/301‑1 (MQ=255)`  
    `aagaAGTTGGACACTGCGATGGCTCATCGTATTCTTGCGGACCTTTCGGACCATGAGCGTCGCTCTCCGCAACTCTATAATGCTATTAACAAACTGTTAGACCGCCACAAGTTCCAGATTGGTAAGTTGCAGCCGGATGTTCACATCTTAGGTGGCCTTGCTGGTGCTCTTGAAGAGTACAAAGAGAAAGTCGGTGATAACGGTCTTACGGATGATGATATTTACACATTACAGTGATATACTCAAGGCCACTACAGATAGTGGTCTTTATGGATGTCATTGTCTATACGAGATG‑CTCCTa                                                                                                                                                                                                                                                                                                          <  1:42711/300‑1 (MQ=255)`  
      `caggTTGGACACTGCGATGGCTCAGCGTATGCTTGCGGACCTTTCGGACCATGAGCGTCGCTCTCAGCAACTCTATAATGCTATTAACAAACTGTTAGACCGCCACAAGTTCCAGATTGGTAAGTTGCAGCCGGATGTTCACATCTTAGGTGGCCTTGCTGGTGCTCTTGAAGAGTACAAAGAGAAAGTCGGTGATAACGGTCTTACGGATGATGATATTTACACATTACAGTGATATACTCAAGGCCACTACAGATAGTGGTCTTTATGGATGTCATTGTCTATACGAGATG‑CTCCTACg                                                                                                                                                                                                                                                                                                        <  1:270031/298‑1 (MQ=255)`  
       `aTGTTGGACACTGCGATGGCTCAGCGTATGCTTGCGGACCTTTCGGACCATGAGCGTCGCTCTCCGCAACTCTATAATGCTCTTAACAAACTGTTAGACCGCCACAAGTTCCAGATTGGTAAGTTGCAGCCGGATGTTCACATCTTAGGTTGCCTTGCTGGTGCTCTTGAAGAGTACAAAGAGAAAGTCGGTGATAACGGTCTTACGGATGATGATATTTACACATTACAGTGATATACTCAAGGCCACTACCGATTGTGGTCTTTATGGATGTCATTGTCTATACGAGATG‑CTCCTACGt                                                                                                                                                                                                                                                                                                       >  1:81786/1‑301 (MQ=255)`  
         `gTTGGACACTGCGATGGCTCAGCGTATGCTTGCGGACCTTTCGGACCATGAGCGTCGCTCTCCGCAACTCTATAATGCTATTAACAAACTGTTAGACCGCCACAAGTTCCAGATTGGTAAGTTGCAGCCGGATGTTCACATCTTAGGTGGCCTTGCTGGTGCTCTTGAAGAGTACAAAGAGAAAGTCGGTGATAACGGTCTTACGGATGATGATATTTACACATTACAGTGATATACTCAAGGCCACTACAGATAGTGGTCTTTATGGATGTCATTGTCTATACGAGATG‑CTCCTACGTGa                                                                                                                                                                                                                                                                                                     >  1:208173/1‑301 (MQ=255)`  
           `aagagacagCGATGGCTCAGCGTATGCTTGCGGACCTTTCGGACCATGAGCGTCGCTCTCCGCAAATCTATAATGCTATTAACAAACTGTTAGACCGCCACAAGTTCCAGATTGGTAAGTTGCAGCCGGATGTTCACATCTTAGGTGGCCTTGCTGGTGCTCTTGAAGAGTACAAAGAGAAAGTCGGTGATAACGGTCTTACGGATGATGATATTTACACATTACAGTGATATACTCAAGGCCACTACAGATAGTGGTCTTTATGGATGTCATTGTCTATACGAGATG‑Ctaaacgtattct                                                                                                                                                                                                                                                                                                   <  1:576803/293‑12 (MQ=255)`  
            `ggACACTGCGATGGCTCAGCGTATGCTTGCGGACCTTTCGGACCATGAGCGTCGCTCTCCGCAACTCTATAATGCTATTAACAAACTGTTAGACCGCCACAAGTTCCAGATTGGTAAGTTGCAGCCGGATGTTCACATCTTAGGTGGCCTTGCTGGTGCTCTTGAAGAGTACAAAGAGAAAGTCGGTGATAACGGTCTTACGGATGATGATATTTACACATTACAGTGATATACTCAAGGCCACTACAGATAGTGGTCTTTATGGATGTCATTGTCTATACGAGATG‑Ctaaacgtattctc                                                                                                                                                                                                                                                                                                  >  1:236303/1‑289 (MQ=255)`  
                 `cTGCGATGGCTCAGCGTATGCTTGCGGACCTTTCGGACCATGAGCGTCGCTCTCCGCAACTCTATAATGCTATTAACAAACTGTTAGACCGCCACAAGTTCCAGATTGGTAAGTTGCAGCCGGATGTTCACATCTTAGGTGGCCTTGCTGGTGCTCTTGAAGAGTACAAAGAGAAAGTCGGTGATAACGGTCTTACGGATGATGATATTTACACATTACAGTGATATACTCAAGGCCACTACAGATAGTGGTCTTTATGGATGTCATTGTCTATACGAGATG‑Ctaaacgtattctagcagt                                                                                                                                                                                                                                                                                             >  1:672912/1‑284 (MQ=255)`  
                   `gCGATGGCTCAGCGTATGCTTGCGGACCTTTCGGACCATGAGCGTCGCTCTCCGCAACTCTATAATGCTATTAACAAACTGTTAGACCGCCACAAGTTCCAGATTGGTAAGTTGCAGCCGGATGTTCACATCTTAGGTGGCCTTGCTGGTGCTCTTGAAGGTTCCAAGGGGAAGTTCGTTAATAAGGGCTTTAGGGAGGAGGATATTTAACCTTTAAGGGGATA‑ACTCAAGGGCCCTACAGGTAGTGGTTTTTTTGGGTGGCAATTGCTATTAGAGATGCCTCCTA‑GGGGAATCTGGAAgg                                                                                                                                                                                                                                                                                          >  1:371034/1‑300 (MQ=255)`  
                     `cagggCTCAGCGTAGGCTTGCGGACCTGTCGGACCATGAGCGTCGCTCTCCGCAACTATATAATGCTATTAACAAACTGTAAGACCGCCACAAGTTCCAGATTGGTAAGTTGCAGCCGGATGTTCACATCTTAGGTGGCCTTGCTGGTGCTCTTGAAGAGTACAAAGAGAAAGTCGGTGATAGCGGTCTTACGGATGATGATATTTACACATTACAGTGATATACTCAAGGCCACTACAGATAGTGGTCTTTATGGATGTCATTGTCTATACGAGATG‑CTCCTACGTGAAATCTGAAAGtt                                                                                                                                                                                                                                                                                         <  1:570567/298‑1 (MQ=255)`  
                               `cGTATGCTTGCGGACCTTTCGGACCATGAGCGTCGCTCTCCGCAACTCTATAATGCTATTAACAAACTGTTAGACCGCCACAAGTTCCAGATTGGTAAGTTGCAGCCGGATGTTCACATCTTAGGTGGCCTTGCTGGTGCTCTTGAAGAGTACAAAGAGAAAGTCGGTGATAACGGTCTTACGGATGATGATATTTACACATTACAGTGATATACTCAAGGCCACTACAGATAGTGGTCTTTATGGATGTCATTGTCTATACGAGATG‑CTCCTACGTGAAATCTGAAAGTTAACGGGAGGc                                                                                                                                                                                                                                                                               <  1:697860/301‑1 (MQ=255)`  
                                   `tGCTTGCGGACCTTTCGGACCATGAGCGTCGCTCTCCGCAACTCTATAATGCTATTAACAAACTGTTAGACCGCCACAAGTTCCAGATTGGTAAGTTGCAGCCGGATGTTCACATCTTAGGTGGCCTTGCTGGTGCTCTTGAAGAGTACAAAGAGAAAGTCGGTGATAACGGTCTTACGGATGATGATATTTACACATTACAGTGATATACTCAAGGCCACTACAGATAGTGGTCTTTATGGATGTCATTGTCTATACGAGATG‑ATCCTACGTGAACTCTGAAAGTTAACGGGAGGCATTa                                                                                                                                                                                                                                                                           >  1:786781/1‑301 (MQ=255)`  
                                       `tGCGGACCTTTCGGACCATGAGCGTCGCTCTCCGCAACTCTATAATGCTATTAACAAACTGTTAGACCGCCACAAGTTCCAGATTGGTAAGTTGCAGCTGGATGTTCACATCTTAGGTGGCCTTGCTGGTGCTCTTGAAGAGTACAAAGAGAAAGTCGGTGATAACGGTCTTACGGATGATGATATTTACACATTACAGTGATATACTCAAGGCAACTACAGATAGTGGTCTTTATGGATGTCATTGTCTATACGAGATG‑CTCCTACGTGAAATCTGAAAGTTAACGGGAGGCATTATGCt                                                                                                                                                                                                                                                                       <  1:621351/301‑1 (MQ=255)`  
                                                       `cATGAGCGTCGCTCTCCGCAACTCTATAATGCTATTAACAAACTGTTAGACCGCCACAAGTTCCAGATTGGTAAGTTGCAGCCGGATGTTCACATCTTAGGTGGCCTTGCTGGTGCTCTTGAAGAGTACAAAGAGAAAGTCGGTGATAACGGTCTTACGGATGATGATATTTACACATTACAGTGATATACTCAAGGCCACTACAGATAGTGGTCTTTATGGATGTCATTGTCTCTACGAGATG‑CTCCTACGTGAACTCTGAAAGTTACCGGGAGGAATTATGCTAGAATTTTTACGTAAg                                                                                                                                                                                                                                                       >  1:354373/1‑301 (MQ=255)`  
                                                              `gTCGCTCTCGGCAACTCTATAATGCTATTAACAAACTGTTAGACCGCCTCAAGTTCCAGGTTGGTAAGTTGCAGCCGGATGTTCACATCTTAGGTGGCCTTGCTGGTGCTCTTGAAGAGTACAAAGAGAAAGTCGGTGATAACGGTCTTACGGATGATGATATGTACACATTACAGTGATATACTCAAGGCCACTACAGATAGTGGTATTTATGGATGTCATTGTCTATACGAGATG‑CTCCTACGTGAAATCTGAAAGTTAACGGGAGGCATTATGATAGAATTTTTACGTAAGCTAATcc                                                                                                                                                                                                                                                <  1:395666/301‑1 (MQ=255)`  
                                                              `gTCGCTCTCCGCAACTCTATAATGCTATTAACAAACTGTTAGACCGCCACAAGTTCCAGATTGGTAAGTTGCAGCCGGATGTTCACATCTTAGGTGGCCTTGCTGGTGCTCTTGAAGAGTACAAAGAGAAAGTCGGTGATAACGGTCTTACGGATGATGATATTTACACATTACAGTGATATACTCAAGGCCACTACAGATAGTGGTCTTTATGGATGTCATTGTCTATACGAGATG‑CTCCTACGTGAAATCTGAAAGTTAACGGGAGGCATTATGCTAGAATTTTTACGTAAGCTAATcc                                                                                                                                                                                                                                                <  1:493830/301‑1 (MQ=255)`  
                                                                           `aCTCTATAATGCTATTAACAAACTGTTAGACCGCCACAAGTTCCAGATTGGTAAGTTGCAGCCGGATGTTCACATCTTAGGTGGCCTTGCTGGTGCTCTTGAAGAGTACAAAGAGAAAGTCGGTGATAACGGTCTTACGGATGATGATATTTACACATTACAGTGATATACTCAAGGCCACTACAGATAGTGGTCTTTATGGATGTCATTGTCTATACGAGATG‑CTCCTACGTGAAATCTGAAAGTTAACGGGAGGCATTATGCTAGAATTTTTACGTAAGCTAATCCCTTGGGTTCTCGc                                                                                                                                                                                                                                   >  1:726757/1‑301 (MQ=255)`  
                                                                           `aCTCTATAATGCTATTAACAAACTGTTAGACCGCCACAAGTTCCAGATTGGTAAGTGGCAGCCGGATGTTCACATCGTAGGTGGCCTTGCTGGGGCTCTTGAAGAGTACAAAGAGAAAGGCGGTGATAACGGTCTTACGGATGATTATATTTACACATTACAGTGATATACTCAAGGCCACTACAGATATTGGTCTTTATGGATGTCATTGTCTATACGAGATG‑CTCCTACGTGAAATCTGAAAGTTAACGGGAGGCATTATGCTAGAATTTTTACGTAAGCTAATCCCTTGGGTTCTCGc                                                                                                                                                                                                                                   <  1:710747/301‑1 (MQ=255)`  
                                                                              `ctATAATGCTATTAACAAACTGTTAGACCGCCACAAGTTCCAGATTGGTAAGTTGCAGCCGGATGTTCACATCTTAGGTGGCCTTGCTGGTGCTCTTGAAGAGTACAAAGAGAAAGTCGGTGATAACGGTCTTACGGATGATGATATTTACACATTACAGTGATATACTCAAGGCCACTACAGATAGTGGTCTTTATGGATGTCATTGTCTATACGAGATG‑CTCCTACGTGAAATCTGAAAGTTAACGGGAGGCATTATGCTAGAATTTTTATGTAAGCTAATCCCTTGGGTTCTCGCTgg                                                                                                                                                                                                                                >  1:795932/1‑301 (MQ=255)`  
                                                                              `ctATAATGCTATTAACAAACTGTTAGACCGCCACAAGTTCCAGATTGGTAAGTTGCAGCCGGATGTTCACATCTTAGGTGGCCTTGCTGGTGCTCTTGAAGAGTACAAAGAGAAAGTCGGTGATAACGGTCTTACGGATGATGATATTTACACATTACAGTGATATACTCAAGGCCACTACAGATAGTGGTCTTTATGGATGTCATTGTCTATACGAGATG‑CTCCTACGTGAAATCTGAAAGTTAACGGGAGGCATTATGCTAGAATTTTTACGTAAGCtctgtctcttatacacatctcc                                                                                                                                                                                                                                >  1:188089/1‑280 (MQ=255)`  
                                                                              `ctATAATGCTATTAACAAACTGTTAGACCGCCACAAGTTCCAGATTGGTAAGTTGCAGCCGGATGTTCACATCTTAGGTGGCCTTGCTGGTGCTCTTGAAGAGTACAAAGAGAAAGTCGGTGATAACGGTCTTACGGATGATGATATTTACACATTACAGTGATATACTCAAGGCCACTACAGATAGTGGTCTTTATGGATGTCATTGTCTATACGAGATG‑CTCCTACGTGAAATCTGAAAGTTAACGGGAGGCATTACGCTAGAATTTTTACGTAAGCTAATCCCTTGGGTTCTCGCTgg                                                                                                                                                                                                                                >  1:298299/1‑301 (MQ=255)`  
                                                                              `ctATAATGCTATTAACAAACTGTTAGACCGCCACAAGTTCCAGATTGGTAAGTTGCAGCCGGATGTTCACATCTTAGGTGGCCTTGCTGGTGCTCTTGAAGAGTACAAAGAGAAAGTCGGTGATAACGGTCTTACGGATGATGATATTTACACATTACAGTGATATACTCAAGGCCACTACAGATAGTGGTCTTTATGGATGTCATTGTCTATACGAGATG‑CTCCTACGTGAAATCTGAAAGTTAACGGGAGGCATTAAGCTAGAATTTTTACGTAAGCTAATCCCTTGGGTTCTCGCTgg                                                                                                                                                                                                                                >  1:277314/1‑301 (MQ=255)`  
                                                                              `ctATAATGCTATTAACAAACTGTTAGACCGCCACAAGTTCCAGATTGGTAAGTTGCAGCCGGATGTTCACATCTCAGGTGGCCTTGCTGGTGCTCTTGAAGAGTACAAAGAGAAAGTCGGTGATAACGGTCTTACGGATGATGATATTTACACATTACAGTGATATACTCAAGGCCACTACAGATAGTGGTCTTTATGGATGTCATTGTCTATAAGAGATG‑CTCCTACGTGAAATCTGAAAGGTAACGGGAGGCATGAGGCTAGAATTTTTACGTAAGCTAATCCATTGGGTTCTCGCtcg                                                                                                                                                                                                                                >  1:771177/1‑299 (MQ=255)`  
                                                                                        `aTTAACAAACTGTTAGACCGCCACAAGTTCCAGATTGGTAAGTTGCAGCCGGATGTTCACATCTTAGGTGGCCTTGCTGGTGCTCTTGAAGAGTACAAAGAGAAAGTCGGTGATAACGGTCTTACGGATGATGATATTTACACATTACAGTGATATACTCAAGGCCACTACAGATAGTGGTCTTTATGGATGTCATTGTCTATACGAGATG‑CTCCTACGTGAAATCTGAAAGTTAACGGGAGGCATTATGCTAGAATTTTTACGTAAGCTAATCCCTTGGGTTCTCGCTGGGATGCTATTc                                                                                                                                                                                                                      >  1:121107/1‑301 (MQ=255)`  
                                                                                           `aaCAAACTGTTAGACCGCCACAAGTTCCAGATTGGTAAGTTGCAGCCGGATGTTCACATCTTAGGTGGCCTTGCTGGTGCTCTTGAAGAGTACAAAGAGAAAGTCGGTGATAACGGTCTTACGGATGATGATATTTACACATTACAGTGATATACTCAAGGCCACTACAGATAGTGGTCTTTATGGATGTCATTGTCTATACGAGATG‑CTCCTACGTGAAATCTGAAAGTTAACGGGAGGCATTATGCTAGAATTTTTACGTAAGCTAATCCCTTGGGTTCTCGCTGGGATGCTATTCggg                                                                                                                                                                                                                   >  1:150780/1‑301 (MQ=255)`  
                                                                                                 `cTGTTAGACCGCCACAAGTTCCAGATTGGTAAGTTGCAGCCGGATGTTCACATCTTAGGTGGCCTTGCTGGTGCTCTTGAAGAGTACAAAGAGAAAGTCGGTGATAACGGTCTTACGGATGATGATATTTACACATTACAGTGATATACTCAAGGCCACTACAGGTCGTGGTCTTTATGGATGTCATTGTCTATACGAGATG‑CTCCTACGTGAAATCTGAAAGTTAACGGGAGGCATTATGCTAGAATTTTTACGTAAGCTAATCCCTTGGGTTCTCGCTCGGATGCTATTCGGGTTAGGa                                                                                                                                                                                                             >  1:195629/1‑301 (MQ=255)`  
                                                                                                  `tGTTAGACCGCCACAAGTTCCAGATTGGTAAGTTGCAGCCGGATGTTCACATCTTAGGTGGCCTTGCTGGTGCTCTTGAAGAGTACAAAGAGAAAGTCGGTGATAACGGTCTTACGGATGATGATATTTACACATTACAGTGATATACTCAAGGCCACTACAGATAGTGGTCTTTATGGATGTCATTGTCTATACGAGATG‑CTCCTACGTGAAATCTGAAAGTTAACGGGAGGCATTACGCTAGAATTTTTACGTAAGCTAATCCCTTGGGTTCTCGCTGGGATGCTATTCGGGTTAGGAt                                                                                                                                                                                                            <  1:65746/301‑1 (MQ=255)`  
                                                                                                       `gACCGCCACAAGTTCCAGATTGGTAAGTTGCAGCCGGATGTTCACATCTTAGGTGGCCTTGCTGGTGCTCTTGAAGAGTACAAAGAGAAAGTCGGTGATAACGGTCTTACGGATGATGATATTTACACATTACAGTGATATACTCAAGGCCACTACAGATAGTGGTCTTTATGGATGTCATTGTCTATACGAGATG‑CTCCTACGTGAAATCTGAAAGTTAACGGGAGGCATTATGCTAGAATTTTTACGTAAGCTAATCCCTTGGGTTCTCGCTGGGATGCTATTCGGGTTAGGATGGCAt                                                                                                                                                                                                       <  1:159285/301‑1 (MQ=255)`  
                                                                                                           `gCCACAAGTTCCAGATTGGTAAGTTGCAGCCGGATGTTCACATCTTAGGTGGCCTTGCTGGTGCTCTTGAAGAGTACAAAGAGAAAGTCGGTGATAACGGTCTTACGGATGATGATATTTACACATTACAGTGATATACTCAAGGCCACTACAGATAGTGGTCTTTATGGATGTCATTGTCTATACGAGATG‑CTCCTACGTGAAATCTGAAAGTTAACGGGAGGCATTATGCTAGAATTTTTACGTAAGCTAATCCCTTGGGTTCTCGCTGGGATGCTATTCGGGTTAGGATGGCATCTAg                                                                                                                                                                                                   <  1:754896/301‑1 (MQ=255)`  
                                                                                                                  `gTTCCAGATTGGTAAGTTGCAGCCGGATGTTCACATCTTAGGTGGCCTTGCTGGTGCTCTTGAAGAGTACAAAGAGAAAGTCGGTGATAACGGTCTTACGGATGATGATATTTACACATTACAGTGATATACTCAAGGCCACTACAGATAGTGGTCTTTATGGATGTCATTGTCTATACGAGATG‑CTCCTACGTGAAATCTGAAAGTTAACGGGAGGCATTATGCTAGAATTTTTACGTAAGCTAATCCCTCGGGTTCTCGCTGGGATGCTATTCGGGTTAGGATGGCATCTAGGGTCAGa                                                                                                                                                                                            >  1:243679/1‑301 (MQ=255)`  
                                                                                                                   `ttCCAGATTGGTAAGTTGCAGCCGGATGTTCACATCTTAGGTGGCCTTGCTGGTGCTCTTGAAGAGTACCAAGAGAAAGTCGGTGATAACGGTCTTACTGATGATGGTATTTACACATTACAGTGATATACTCAAGTCCACTACAGATAGTGGTCTTTATGGATGTCATTGTCTATACGAGATG‑CTCCTACGTGAAATCTGAAAGTTAACTGGAGGCATTATGCTAGAATTTTTACGTAAGCTAATCCCTTGGGTTCTCGCTGGGATGCTATTCGGGTTAGGATGGCATCTAGGGTCAGAc                                                                                                                                                                                           <  1:680117/301‑1 (MQ=255)`  
                                                                                                                   `ttCCAGATTGGTAAGTTGCAGCCGGATGTTCACATCTTAGGTGGCCTTGCTGGTGCTCTTGAAGAGTACAAAGAGAAAGTCGGTGATAACGGTCTTACGGATGATGATATTTACACATTACAGTGATATACTCAAGGCCACTACAGATAGTGGTCTTTATGGATGTCATTGTCTATACGAGATG‑CTCCTACGTGAAATCTGAAAGTTAACGGGAGGCATTATGCTAGAATTTTTACGTAAGCTAATCCCTTGGGTTCTCGCTGGGATGCTATTCGGGTTAGGATGGCATCTAGGGTCAGAc                                                                                                                                                                                           <  1:257660/301‑1 (MQ=255)`  
                                                                                                                         `aTTGGTAAGTTGCAGCCGGATGTTCACATCTTAGGTGGCCTTGCTGGTGCTCTTGAAGAGTACAAAGAGAAAGTCGGTGATAACGGTCTTACGGATGATGATATTTACACATTACAGTGATATACTCAAGGCCACTACAGATAGTGGTCTTTATGGATGTCATTGTCTATACGAGATG‑CTCCTACGTGAAATCTGAAAGTTAACGGGAGGCATTACGCTAGAATTTTTACGTAAGCTAATCCCTTGGGTTCTCGCTGGGATGCTATTCGGGTTAGGATGGCATCTAGGGTCAGACTCAATg                                                                                                                                                                                     <  1:378527/301‑1 (MQ=255)`  
                                                                                                                                        `ctcggagatgtgtataagagacagcTTGCTGGTGCTCTTGAAGAGTACAAAGAGAAAGTCGGTGATAACGGTCTTACGGATGATGATATTTACACATTACAGTGATATACTCAAGGCCACTACAGATAGTGGTCTTTATGGATGTCATTGTCTATACGAGATG‑CTCCTACGTGAAATCTGAAAGTTAACGGGAGGCATTATGCTAGAATTTTTACGTAAGCTAATCCCTTGGGTTCTCGCTGGGATGCTATTCGGGTTAGGATGGCATCTAGGGTCAGACTCAATGGACGCTAAATGGaaa                                                                                                                                                                      <  1:403467/277‑1 (MQ=255)`  
                                                                                                                                            `aTGTTAACATCTTAGGTGGCCTTGCTGGTGCGCTTGAAGAGTACAAAGAGAAAGTCGGTGATAACGGTCTTACGGATGATGATATTTACACATTACAGTGATATACTCAAGGCCACTACAGATAGTGGTCTTTATGGATGTCATTGTCTATACGAGATG‑CTCCTACGTGAAATCTGAAAGTTAACGGGAGGCATTATGCTAGAATTTTTACGTAAGCTAATCCCTTTGGTTCTCGCTGGGATGCTATTCGGGTTAGGATGGCATCTAGGGTCAGACTCAATGGACGCTAAATGGAAACagg                                                                                                                                                                  <  1:470059/301‑1 (MQ=255)`  
                                                                                                                                             `tGTTCACATCTTAGGTGGCCTTGCTGGTGCTCTTGAAGAGTACAAAGAGAAAGTCGGTGATAACGGTCTTACGGATGATGATATTTACACATTACAGTGATATACTCAAGGCCACTACAGATAGTGGTCTTTATGGATGTCATTGTCTATACGAGATG‑CTCCTACGTGAAATCTGCAAGTTAACGGGAGGCATTACGCTAGAATTTTTACGTAAGATAATACCTTGGGTTCGCGCTGGGATGCTATTCGGGTTAGGATGGCATCTAGGATCAGACTAAACGCACGCCAAATGGAACAagga                                                                                                                                                                 >  1:315599/1‑301 (MQ=255)`  
                                                                                                                                               `ttCACATCTTAGGTGGCCTTGCTGGTGCTCTTGAAGAGTACAAAGAGAAAGTCGGTGATAACGGTCTTACGGATGATGATATTTACACATTACAGTGATATACTCAAGGCCACTACAGATAGTGGTCTTTATGGATGTCATTGTCTATACGAGATG‑CTCCTACGTGAAATCTGAAAGTTAACGGGAGGCATTATGCTAGAATTTTTACGTAAGCTAATCCCTTGGGTTCTCGCTGGGATGCTATTCGGGTTAGGATGGCATCTAGGGTCAGACTCAATGGACGCTAAATGGAAACaggagg                                                                                                                                                               <  1:214703/301‑1 (MQ=255)`  
                                                                                                                                                 `gagacagtAGGTGGCCTTGCTGGTGCTCATGAAGAGTACAAAGAGAAAGTCGGTGATAACGGTCTTACGGATGATGATATTTACACATTACAGTGATATACTCAAGGCCACTACAGATAGTGGTCTTTATGGATGTCATTGTCTATACGAGATG‑CTCCTACGTGAAATCTGAAAGTTAACGGGAGGCATTATGCTAGAATTTTTACGTAAGCTAATCCCTTGGGTTCTCGCTGGGATGCTATTCGGGTTAGGATGGCATCTAGGGTCAGACTCAATGGACGCTAAATGGAAACAGGAGGTa                                                                                                                                                             <  1:463616/294‑1 (MQ=255)`  
                                                                                                                                                   `caTCTTAGGTGGCCTTGCTGGTGCTCTTGAAGAGTACAAAGAGAAAGTCGGTGATAACGGTCTTACGGATGATGATATTTACACATTACAGTGATATACTCAAGGCCACTACAGATAGTGGTCTTTATGGATGTCATTGTCTATACGAGATG‑CTCCTACGTGAAATCTGAAAGTTAACGGGAGGCATTACGCTAGAATTTTTACGTAAGCTAATCCCTTGGGTTCTCGCTGGGATGCTATTCGGGTTAGGATGGCATCTAGGGTCAGACTCAATGGACGCTAAATGGAAACAGGAGGTaca                                                                                                                                                           >  1:734741/1‑301 (MQ=255)`  
                                                                                                                                                        `ggggttcggagatgtgtataagagacaggTACAAAGAGAAAGTCGGTGATAACGGTCTTACGGATGATGATATTTACACATTACAGTGATATACTCAAGGCCACTACAGATAGTGGTCTTTATGGATGTCATTGTCTATACGAGATG‑CTCCTACGTGAAATCTGAAAGTTAACGGGAGGCATTATGCTAGAATTTTTACGTAAGCTAATCCCTTGGGTTCTCGCTGGGATGCTATTCGGGTTAGGATGGCATCTAGGGTCAGACTCAATGGACGCTAAATGGAAACAGGAGGTACACAATg                                                                                                                                                      <  1:476719/273‑1 (MQ=255)`  
                                                                                                                                                        `ggacaggCCTTGCTGGTGCTCTTGAAGAGTACAAAGAGAAAGTCGGTTATAACGGTCTTACGGATGATGATATTTACACATTACAGTGATATACTCAAGGCCACTACAGATAGTGGTCTTTATGGATGTCATTGTCTATACGAGATG‑CTCCTACGTGAAATCTGAAAGTTAACGGGAGGCATTATGCTAGAATTTTTACGTAAGCTAATCCCTTGGGTTCTCGCTGGGATGCTATTCGGGTTAGGATGGCATCTAGGGTCAGACTCAATGGACGCTAAATGGAAACAGGAGGTACACAATg                                                                                                                                                      <  1:58295/296‑1 (MQ=255)`  
                                                                                                                                                            `gggCCTGGCTGGTGCTCTTGAAGAGTAAAAAGTGAAATCCGGTGATAACTGTCTTACGGATGATGATATTGACACATTACAGTGATATACTCAAGGACACTACAGATAGTGGTCTTTATGGATGTCATGGTCTATACGAGATG‑CTCCTACGTGAACTCTGAAAGTTCACGGGAGGCATTACGCTAGAATTTTTACGTAAGCTAATCCCTTGGGTTCTCGCTGGGATGCTATTCGGGTTAGGATGGCATCTAGGGTCAGACTCAATGGACGCTAAATGGAAACAGGAGGTACACAATGAGTa                                                                                                                                                  <  1:595973/300‑1 (MQ=255)`  
                                                                                                                                                                `cTTGCTGGTGCTCTTGAAGAGTACAAAGAGAAAGTCGGTGATAACGGTATTACGGATGATGATATTTACACATTACAGTGATATACTCAAGGCCACTACAGATAGTGGTCTTTATGGATGTCATTGTCTATACGAGATG‑CTCCTACGTGAAATCTGAAAGTTAACGGGAGGCATTACGCTAGAATTTTTACGTAAGCTAAGCCCTTGGGTTCTCGATGGGATGATATTCGGGTTAGGATGGCATCTAGGGTCAGACTCAATGGACGCTAAATGGAAACAGGAGGTACACAATGAGTACGtt                                                                                                                                              <  1:4850/301‑1 (MQ=255)`  
                                                                                                                                                                     `tGGTGCTCTTGAAGAGTACAAAGAGAAAGTCGGTGATAACGGTCTTACGGATGATGATATTTACACATTACAGTGATATACTCAAGGCCACTACAGATAGTGGTCTTTATGGATGTCATTGTCTATACGAGATG‑CTCCTACGTGAAATCTGAAAGTTAACGGGAGGCATTATGCTAGAATTTTTACGTAAGCTAATCCCTTGGGTTCTCGCTGGGATGCTATTCGGGTTAGGATGGCATCTAGGGTCAGACTCAATGGACGCTAAATGGAAACAGGAGGTACACAATGAGTACGTTAagag                                                                                                                                         <  1:628406/301‑1 (MQ=255)`  
                                                                                                                                                                     `aaagagacaggAAGAGGACAAAGAGAAAGTCGGTGATAACGGTCTTACGGATGATGATATTTACACATTACAGTGATATACTCAAGGCCACTACAGATAGTGGTCTTTATGGATGTCATTGTCTATACGAGATG‑CTCCTACGTGAAATCTGAAAGTTAACGGGAGGCATTACGCTAGAATTTTTACGTAAGCTAATCCCTTGGGTTCTCGCTGGGATGCTATTCGGGTTAGGATGGCATCTAGGGTCAGACTCAATGGACGCTAAATGGAAACAGGAGGTACACAATGAGTACGTTAagag                                                                                                                                         <  1:590378/291‑1 (MQ=255)`  
                                                                                                                                                                       `gTGCTCTTGAAGAGTACAAAGAGAAAGTCGGTGATAACGGTCTTACGGATGATGATATTTACACATTACAGTGATATACTCAAGGACACTACAGATAGTGGTCTTTATGGATGTCATTGTCTATACGAGATG‑CTCCTCCGTGAAATCTGAAAGTTAACGGGAGGCATTATGCTAGAATTTTTACGTAAGCTAATCCCTTGGGTTCTCGCTGGGATGCTATTCGGGTTAGGATGGCATCTAGGGTCAGACTCAATGGACGCAAAATGGAAACAGGAGGTACACAATGAGTACGTTAagagag                                                                                                                                       >  1:340201/1‑301 (MQ=255)`  
                                                                                                                                                                       `gTGCTCTTGAAGAGTACAAAGAGAAAGTCGGGGATAACGGTCTTACGGATGATGATATTTACACATTACAGTGATATACTCAAGGCCACTACAGATAGTGGTCTTTATGGATGTCATTGTCTCTACGAGATG‑CTCCTACGTGAAATCTGAAAGTTAACGGGAGGCATTACGCTAGAATTTTTACGTAAGCTAATCCCTTGGGTTCTCGCTGGGATGCTATTCGGGTTAGGATGGCATCTAGGGTCAGACTCAATGGACGCTAAATGGAAACAGGAGGTACACAATGAGTACGTTAagagag                                                                                                                                       <  1:363114/301‑1 (MQ=255)`  
                                                                                                                                                                          `ctctTGAAGAGTACAAAGAGAAAGTCGGTGATAACGGTCTTACGGATGATGATATTTACACATTACAGTGATATACTCAAGGCCACTACAGATAGTGGTCTTTATGGATGTCATTGTCTATACGAGATG‑CTCCTACGTGAAATCTGAAAGTTAACGGGAGGCATTATGCTAGAATTTTTACGTAAGCTAATCCCTTGGGTTCTCGCTGGGATGCGATTCGGGTTAGGATGGCATCGAGGGTCAGACTCAATGGACGCGAAATGGAAACAGGAGGTACAAAATGAGTACGGTAAGAGAGTTg                                                                                                                                    >  1:718428/1‑301 (MQ=255)`  
                                                                                                                                                                          `ctctTGAAGAGTACAAAGAGAAAGTCGGTGATAACGGTCTTACGGATGATGATATTTACACATTACAGTGATATACTCAAGGCCACTACAGATAGTGGTCTTTATGGATGTCATTGTCTATACGAGATG‑CTCCTACGTGAAATCTGAAAGTTAACGGGAGGCATTACGCTAGAATTTTTACGTAAGCTAATCCCTTGGGTTCTCGCTGGGATGCTATTCGGATTAGGATGGCATCTAGGGTCAGACTCAATGGACGCTAAATGGAAACAGGAGGTACACCATGAGTACGTTAAGAGAGTTg                                                                                                                                    >  1:412012/1‑301 (MQ=255)`  
                                                                                                                                                                          `ctctTGAAGAGTAAAAAGAGAAAGTCGGTGATAACGGTCTTACGGATGATGATATTTACACATTACAGTGATATACTCAAGGCCACTACAGATAGTGGTCTTTATGGATGTCATTGTCTATACGAGATG‑CTCCTACGTGAAATCTGAAAGTTAACGGGAGGCATTACGATAGAATTTTTACGTAAGCTAATCCCTTGGGTTCTCGCTGGGATGCTATTCGGGTTAGGATGGCATCTAGGGTCAGACTCAATGGACGCTAAATGGAAACAGGAGGTACACAATGAGTACGTTAAGAGAGTTg                                                                                                                                    <  1:84974/301‑1 (MQ=255)`  
                                                                                                                                                                            `ctTGAAGAGTACAAAGAGAAAGTCGGTGATAACGGTCTTACGGATGATGATATTTACACATTACAGTGATATACTCAAGGCCACTACAGATAGTGGTCTTTATGGATGTCATTGTCTATACGAGATG‑CTCCTACGTGAAATCTGAAAGTTAATGGGAGGCATTATGCTAGAATTTTTACGTAAGCTAATCCCTTGGGTTCTCGCTGGGATGCTATTCGGGTTAGGATGGCATCTAGGGTCAGACTCAATGGACGCTAAATGGAAACAGGAGGTACACAATGAGTACGTTAAGAGAGTTGAg                                                                                                                                  >  1:181033/1‑301 (MQ=255)`  
                                                                                                                                                                                   `agTACAAAGAGAAAGTCGGTGATAACGGTCTTACGGATGATGATATTTACACATTACAGTGATATACTCAAGGCCACTACAGATAGTGGTCTTTATGGATGTCATTGTCTATACGAGATG‑CTCCTACGTGAAATCTGAAAGTTAACGGGAGGCATTATGCTAGAATTTTTACGTAAGCTAATCCCTTGGGTTCTCGCTGGGATGCTATTCGGGTTAGGATGGCATCTAGGGTCAGACTCAATGGACGCTAAATGGAAACAGGAGGTACACAATGAGTACGTTAAGAGAGTTGAGGCTTCGa                                                                                                                           >  1:63612/1‑301 (MQ=255)`  
                                                                                                                                                                                    `gTACAAAGAGAAAGTCGGTGATAACGGTCTTACGGATGATGATATTTACACATTACAGTGATATACTCAAGGCCACTACAGATAGTGGTCTTTATGGATGTCATTGTCTATACGAGATG‑CTCCTACGTGAAATCTGAAAGTTAACGGGAGGCATTACGCTAGAATTTTTACGTAAGCTAATCCCTTGGGTTCTCGCTGGGATGCTATTCGGGTTAGGATGGCATCTAGGGTCAGACTCAATGGACGCTAAATGGAAACAGGAGGTACACACTGAGTACGTTAAGAGAGTTGAGGCTGCGaa                                                                                                                          >  1:547234/1‑301 (MQ=255)`  
                                                                                                                                                                                    `gTACAAAGAGAAAGTCGGTGATAACGGTCTTACGGATGATGATATTTACACATTACAGTGATATACTCAAGGCCACTACAGATAGTGGTCTTTATGGATGTCATTGTCTATACGAGATG‑CTCCTACGTGAAATCTGAAAG‑TAACGGGAGGCATTATGCTAGAATTTTTACGTAAGCTAATCCCTTGGGTTCTCGCTGGGATGCTATTCGGGTTAGGATGGCATCTAGGGTCAGACTCAATGGACGCTAAATGGAAACAGGAGGTACACAATGAGTACGTTAAGAGAGTTGAGGCTGCGAag                                                                                                                         >  1:779241/1‑301 (MQ=255)`  
                                                                                                                                                                                           `gagaAAGTCGGTGATAACGGTCTTACGGATGATGATATTTACACATTACAGTGATATACTCAAGGCCACTACAGATAGTGGTCTTTATGTATGTCATTGTCTATACGAGATG‑CTCCTACGTGAAATCTGAAAGTTAACGGGAGGCATTACGCTAGAATTTTTACGTAAGCTAATCCCTTGGGTTCTCGCTGGGATGCTATTCGGGTTAGGATGGCATCTAGGGTCAGACTCAATGGACGCTAAATGGAAACAGGAGGTACACAATGAGTACGTTAagagctgtctcttatacacatctccc                                                                                                                   >  1:613058/1‑279 (MQ=255)`  
                                                                                                                                                                                                  `tCGGTGATATCGGTCTTTAGACTGATGGTATTCACCCCCTACAGTGATAGACTCAAGGCCACTACAGATAGTTGTCTTTATCGATTTCATTGTCTATCCGAGATG‑CTCCTACGGGAAATCTGAAAGTTAACGGGAGGCATTACGCTAGAATTTTTACGTAAGCTAATCCCTTGGGTTCTCGCTGGACTGCTATTCGGGTTAGGATGGCATCTAGGGTCAGACTCAATGGACGCTAAATGGAATCAGGAGGTACACAATGAGTACGTTAAGAGAGTTGAGGCTGCGAAGAGCACTCAAagag                                                                                                            <  1:500762/301‑1 (MQ=255)`  
                                                                                                                                                                                                                      `gatgatgatATTTACACATTACAGTGATATACTCAAGGCCACTACAGATAGTGGTCTTTATGGATGTCATTGTCTATACGAGATG‑CTCCTACGTGAAATCTGAAAGTTAACGGGAGGCATTATGCTAGAATTTTTACATAAGCTAATCCCTTGGGTTCTCGCTGGGATGTTATTCGGGTTAGGATGGCATCTAGTGTCAGACTCAATGGACGCTAAATGGAAACAGGAGGTACACAATGAGTACGTTAAGAGAGTTGAGGCTGCGAAGAGCACTCAAAGAGCAATCGCTgtctcttataca                                                                                        >  1:347998/1‑290 (MQ=255)`  
                                                                                                                                                                                                                      `gatgatgatATTTACACATTACAGTGATATACTCAAGGCCACTACAGATAGTGGTCTTTATGGATGTCATTGTCTATACGAGATG‑CTCCTACGTGAAATCTGAAAGTTAACGGGAGGCATTACGCTAGAATTTTTACGTAAGCTAATCCCTTGGGTTCTCGCTGGGATGCTATTCGGGTTAGGATGGCATCTAGGGTCAGACTCAATGGACGCTAAATGGAAACAGGAGGTACACAATGAGTACGTTAAGAGAGTTGAGGCTGCGAAGAGCACTCAAAGAGCAATCGATGCGGTactgtct                                                                                        >  1:251177/1‑295 (MQ=255)`  
                                                                                                                                                                                                                          `gtgatATTTACACATTACAGTGATATACTCAAGGCCACTACAGATAGTGGTCTTTATGGATGTCATTGTCTATACGAGATG‑CTCCTACGTGAAATCTGAAAGTTAACGGGAGGCATTATGCTAGAATTTTTACGTAAGCTAATCCCTTGGGTTCTCGCTGGGATGCTATTCGGGTTAGGATGGCATCTAGGGTCAGACTCAATGGACGCTAAATGGAAACAGGAGGTACACAATGAGTACGTTAAGAGAGTTGAGGCTGCGAAGAGCACTCAAAGAGCAATCGATGCGGTATCTGCTAAGt                                                                                    <  1:508837/300‑1 (MQ=255)`  
                                                                                                                                                                                                                          `aataagagaaaacaTAACAATGATATACTCAAGGCCACTACAGATAGTGGGCTTTATGGATGTCATTGTCTATACGAGATG‑CTCCTACTTGAAATCTGAAAGTTAACGGGAGGCATTACGATAGAATTTTTACGTAAGCTAATCCCTTGGGTGCTCGCTGGGATGCTAATCGGGTTAGGATGGCATCTAGGGTCAGACTCAATGGACGCTAAAGGGAAACAGGAGGTACACAATGAGTACGTTAAGAGAGTTGAGGCTGCGAAGAGCACTCAAAGAGCAATCGATGCGGTATCTGCTAAGt                                                                                    <  1:107542/290‑1 (MQ=255)`  
                                                                                                                                                                                                                            `gatATTTACACATTACAGTGATATACTCAAGGCCACTACAGATAGTGGTCTTTATGGATGTCAATGTCTATACGAGATG‑CTCCTACGTGAAATCTGAAAGTTAACTGGAGGCATTATGCTAGAATTTTTACGTAAGCTAATCCCTTGGGTTCTCGCTGGGATGCTATTCGGGTTAGGATGGCATCTAGGGTCAGACTCAATGGACGCTAAATGGAAACAGGAGGTACACAATGAGTACGTTAAGAGAGTTGAGGCTGCGAAGAGCACTCAAAGAGCAATCGATGCGGTATCTGCTAAGTAt                                                                                  <  1:201947/301‑1 (MQ=255)`  
                                                                                                                                                                                                                              `tatTTACACATTACAGTGATATACTCAAGGCCACTACAGATAGTGGTCTTTATGGATGTCATTGTCTATACGAGATG‑CTCCTACGTGAAATCTGAAAGTTAACGGGAGGCATTATGCTAGAATTTTTACGTAAGCTAATCCCTTGGGCTCTCGCTGGGCTGCTTTTCGGGTTAGGATGGCATCTAGGGTCATCCTCAATGGACGCTAAATGGAAACAGTAGGTACACAATGAGTACGTTAAGAGAGTTGCGGCTGCGAACAGCACTCAAAGAGCAATCGATGCGTTATCTGCTAAGTATCa                                                                                >  1:308012/1‑301 (MQ=255)`  
                                                                                                                                                                                                                              `tatTTACACATTACAGTGATATACTCAAGGCCACTACAGATAGTGGTCTTTATGGATGTCATTGTCTATACGAGATG‑CTCCTACGTGAAATCTGAAAGTTAACGGGAGGCATTACGCTAGAATTTTTACGTAAGCTAATCCCTTGGGTTCTCGCTGGGATGCTATTCGGGTTAGGATGGCATCTAGGGTCAGACTCAATGGACGCTAAATGGAAACAGGAGGTACACAATGAGTACGTTAAGAGAGTTGAGGCTGCGAAGAGCACTCAAAGAGCAATCGATGCGGTATCTGCTAAGTATCa                                                                                >  1:82334/1‑301 (MQ=255)`  
                                                                                                                                                                                                                                 `ttACACATTACAGTGATATACTCATGGCCACTACAGATAGTGGTCTTTATGGATGTCATTGTCTATACGAGATG‑CTCCTACGTGAAATCTGAAAGTTAACGGGAGGCATTATGCTAGAATTTTTACGTAAGCTAATCCCTTGGGTTCTCGCTGGTATGCTATTCGGGTTAGGATGGCATCTAGTGTCAGACTCAATGGACGCTAAATGGAAACAGGAGGTACACAATGAGTACGTTAAGAGAGTTGAGGCTGCGAAGAGCACTCAAAGAGCAATCGATGCGGTATCTGCTAAGTATCaaga                                                                             <  1:448400/301‑1 (MQ=255)`  
                                                                                                                                                                                                                                 `ttACACATTACAGTGATATACTCAAGGCCACTACAGATAGTGGTCTTTATGGATGTCATTGTCTATACGAGATG‑CTCCTACGTGAAATCTGAAAGTTAACGGGAGGCATTACGCTAGAATTTTTACGTAAGCTAATCCCTTGGGTTCTCGCTGGGATGCTATTCGGGTTAGGATGGCATCTAGGGTCAGACTCAATGGACGCTAAATGGAAACAGGAGGTACACAATGAGTACGTTAAGAGAGTTGAGGCTGCGAAGAGCACTCAAAGAGCAATCGATGCGGTATCTGCTAAGTATCaaga                                                                             <  1:322195/301‑1 (MQ=255)`  
                                                                                                                                                                                                                                 `ttACACATTACAGTGAGATACTCAAGTCCACTACAGATAGATGTCTTTATGGATGTCATTGTCTATACGAGATG‑CTCCTACGTGAAATCTGAAAGTTAACGGGAGGCATTATGCTAGAATTTTTACGTAAGCTAATCCCTTGGGTTCTCGCTGGGATGCTATTCGGGTTAGGATGGCATCTAGGGTCAGACTCAATGGACGCTAAATGGAAACAGGAGGTACACAATGAGTACGTTAAGAGAGTTGAGGCTGCGAAGAGCACTCAAAGAGCAATCGATGCGGTATCTGCTAAGTATCaaga                                                                             <  1:540167/301‑1 (MQ=255)`  
                                                                                                                                                                                                                                 `tgtataagagacaggATATACTCAAGGCCACTACAGATAGTGGTCTTTATGGATGTCATTGTCTATACGAGATG‑CTCCTACGTGAAATCTGAAAGTTAACGGGAGGCATTATGCTAGAATTTTTACGTAAGCTAATCCCTTGGGTTCTCGCTGGGATGCTATTCGGGTTAGGATGGCATCTAGGGTCAGACTCAATGGACGCTAAATGGAAACAGGAGGTACACAATGAGTACGTTAAGAGAGTTGAGGCTGCGAAGAGCACTCAAAGAGCAATCGATGCGGTATCTGCTAAGTATCaaga                                                                             <  1:809936/287‑1 (MQ=255)`  
                                                                                                                                                                                                                                    `cacaTTACAGTGATATACTCAAGGCCACTACAGATAGTGGTCTTTATGGATGTCATTGTCTATACGAGATG‑CTTCTACGTGAAATCTGAAAGTTAACGGGATGCATTATGCTAGAATTTTTACGTAAGCTAATCCCTTGGGTTCTCGCTTGGATGCTATTCGGGTTAGGATGGCATCTAGGGTCAGACTCAGTGGACGCTAAATGGAAACAGGAGGTAAACATTGCGTACGTTAAGAGAGTTGAGGCTGCGAAGAGCACTCAACGAGCAATCGCTGCGGTATCTCCTAAGTATCAtgaaga                                                                          >  1:441582/1‑301 (MQ=255)`  
                                                                                                                                                                                                                                     `gtataagagacagataCTCAAGGCCACTACAGATAGTGGTCTTTATGGATGTCATTGTCTATACGAGATG‑CTCCTACGTGAAATCTGAAAGTTAACGGGAGGCATTACGCTAGAATTTTTACGTAAGCTAATCCCTTGGGTTCTCGCTGGGATGCTATTCGGGTTAGGATGGCATCTAGGGTCAGACTCAATGGACGCTAAATGGAAACAGGAGGTACACAATGAGTACGTTAAGAGAGTTGAGGCTGCGAAGAGCACTCAAAGAGCAATCGATGCGGTATCTGCTAAGTATCAAGAAGAc                                                                         <  1:115039/288‑1 (MQ=255)`  
                                                                                                                                                                                                                                      `caTTATAGTGATATACTCAAGGCCACTACAGATAGTGGTCTTTATGGATGTCATTGTCTATACGAGATG‑CTCCTACGTGAAATCTGAAAGTTAACGGGAGGCATTATGCTAGAATTTTTACGTAAGCTAATCCCTTGGGTTCTCGCTGGGATGCTATTCGGGTTAGGATGGCATCTAGGGTCAGACTCAATGGACGCTAAATGGAAACAGGAGGTACACAATGAGTAAGTTAAGAGAGTTGAGGCTGCGAAGAGCACTCAAAGAGCAATCGATGCGGTATCTGcctgtctcttatacacat                                                                        >  1:804116/1‑284 (MQ=255)`  
                                                                                                                                                                                                                                        `ttACAGTGATATACTCAAGGCCACTACAGATAGTGGTCTTTATGGATGTCATTGTCTATACGAGATG‑CTCCTACGTGAAATCTGAAAGTTAACGGGAGGCATTATGCTAGAATTTTTACGTAAGCTAATCCCTTGGGTTCTCGCTGGGATGCTATTCGGGTTAGGATGGCATCTAGGGTCAGACCCAATGGACGCTAAATGGAAACAGGAGGTACACAATGAGTACGTTAAGAGAGTTGAGGCTGCGAAGAGCACTCAAAGAGCAATCGATGCGGTATCTGCTAAGTATCAAGAAGACCtt                                                                      >  1:135356/1‑301 (MQ=255)`  
                                                                                                                                                                                                                                        `agaCAGTGATATACTCAAGGCCACTACAGATAGTGGTCTTTATGGATGTCATTGTCTATACGAGATG‑CTCCTACGTGAAATCTGAAAGTTAACGGGAGGCATTATGCTAGAATTTTTACGTAAGCTAATCCCTTGGGTTCTCGCTGGGATGCTATTCGGGTTAGGATGGCATCTAGGGTCAGACTCAATGGACGCTAAATGGAAACAGGAGGTACACAATGAGTACGTTAAGAGAGTTGAGGCTGCGAAGAGCACTCAAAGAGCAATCGATGCGGTATCTGCTAAGTATAAAGAAGACCtt                                                                      <  1:421375/299‑1 (MQ=255)`  
                                                                                                                                                                                                                                           `aaGTGATATACTCAAGGCCACTACAGATAGTGGTATTTATGGATGTCATTGGCTATACGAGATG‑CTCCTACGTGAAATCTGAAAGTTAACGGGAGGCATTATGCTAGAATTTTTACGTAAGCTAATCCCTTGGGTTCTCGCTGGGATGCTATTCGGGTTAGGATGGCATCTAGGGTCAGACTCAATGGACGCTAAATGGAAACAGGAGGTACACAATTAGTACGTTAAGAGAGTTGAGGCTGCGAAGAGCACTCAAAGAGCAATCGATGCGGTATCTGCTAAGTATCAAGAAGACCTTGcc                                                                   <  1:221233/300‑1 (MQ=255)`  
                                                                                                                                                                                                                                               `gATATACTCAAGGCCACTACAGATAGTGGTCTTTATGGATGTCGTTGTCTATACGAGATG‑CTCCTACGTGAAATCTGAAAGTTAACGGGAGGCATTATGCTAGAATTTTTACGTAAGCTAATCCCTTGGGTTCTCGCTGGGATGCTATTCGGGTTAAGATGGCATCTAGGGTCAGACTCAATGGACGCTAAATGTAAACAGGAGGTACACAATGAGTACGTTAAGAGAGTTGAGGCTGCGAAGAGCACTCAAAGAGCAAACGATGCGGTATCTGCTAAGTATCaagacggtctcttataca                                                               >  1:173461/1‑287 (MQ=255)`  
                                                                                                                                                                                                                                                         `taggctcggagatgtgtataagagacagATGTCATTGTCTATACGAGATG‑CTCCTACGTGAAATCTGAAAGTTAATGGGAGGCATTATGCTAGAATTTTTACGTAAGCTAATCCCTTGGGTTCTCGCTGGGATGCTATTCGGGTTAGGATGGCATCTAGGGTCAGACTCAATGGACGCTAAATGGAAACAGGATGTACACAATGAGTACGTTAAGAGAGTTGAGGCTGCGAAGAGCACTCAAAGAGCAATCGATGCGGTATCTGCTAAGTATCAAGAAGACCTTGCCGCGCTGGAAGGGAg                                                     <  1:229393/274‑1 (MQ=255)`  
                                                                                                                                                                                                                                                         `aGGCCACTACAGATAGTGGTCTTTATGGATGTCATTGTCTATACGAGATG‑CTCCTACGTGAAATCTGAAAGTTAACGGGAGGCATTATGCTAGAATTTTTACGTAAGCTAATCCCTTGGGTTCTCGCTGGGATGCTATTCGGGTTAGGATGGCATCTAGGGTCAGACTCAATGGACGCTAAATGGAAACAGGAGGTACACAATGAGTACGTTAAGAGAGTTGAGGCTGCGAAGAGCACTCAAAGAGCAATCGATGCGGTATCTGCTAAGTATCAAGAAGACCTTGCCGCGCTGGAAGGGAg                                                     >  1:748163/1‑301 (MQ=255)`  
                                                                                                                                                                                                                                                          `ggCCACTACAGATAGTGGTCTTTATGGATGTCATTGTCTATACGAGATG‑CTCCTACGTGAAATCTGAAAGTTAACGGGAGGCATTATGCTAGAATTTTTACGTAAGCTAATCCCTTGGGTTCTCGCTGGGATGCTATTCGGGTTAGGATGGCATCTAGGGTCAGACTCAATGGACGCTAAATGGAAACAGGAGGTACACAATGAGTACGTTAAGAGAGTTGAGGCTGCGAAGAGCACTCAAAGAGCAATCGATGCGGTATCTGCTAAGTATCAAGAAGACCtgtctcttatacacatctcc                                                    >  1:741736/1‑282 (MQ=255)`  
                                                                                                                                                                                                                                                          `ggCCACTACAGATAGTGGTCTTTATGGATGTCATTGTCTATACGAGATG‑CTCCTACGTGAAATCTGAAAGTTAACGGGAGGCATTACGCTAGAATTTTTACGTAAGCTAATCCCTTGGGTTCTCGCTGGGATGCTATTCGGGTTAGGATGGCATCTAGGGTCAGACTCAATGGACGCTAAATGGAAACAGGAGGTACACAATGAGTACGTTAAGAGAGTTGAGGCTGCGAAGAGCACTCAAAGAGCAATCGATGCGGTATCTGCTAAGTATCAAGAAGACCTTGCCGCGCTGGAAGGGAGc                                                    >  1:330628/1‑301 (MQ=255)`  
                                                                                                                                                                                                                                                            `ccACTACAGATAGTGGTCTTTATGGATGTCATTGTCTATACGAGATG‑CTCCTACGTGAAATCTGAAAGTTAACGGGAGGCATTATGCTAGAATTTTTACGTAAGCTAATCCCTTGGGTTCTCGCTGGGATGCTATTCGGGTTAGGATGGCATCTAGGGTCAGACTCAATGGACGCTAAATGGAAACAGGAGGTACACAATGAGTACGTTAAGAGAGTTGAGGCTGCGAAGAGCACTCAAAGAGCAATCGATGCGGTATCTGCTAAGTATCaagactgtctcttatacacatctccgagccc                                                  >  1:289772/1‑274 (MQ=255)`  
                                                                                                                                                                                                                                                                   `agagacaggTCTTTATGGATGTCATTGTCTATACGAGATG‑CTCCAACGTGAAATCTGAAAGTTAACGGGAGGCATTATGCTAGAATTTTTACGTAAGCTAATCCCTTGGGTTCTCGCTGGGATGCTATTCGGGTTAGGATGGCATCTAGGGTCAGACTCAATGGACGCTAAATGGAAACAGGAGGTACACAATGAGTACGTTAAGAGAGTTGAGGCTGCGAAGAGCACTCAAAGAGCAATCGATGCGGTATCTGCTAAGTATCAAGAAGACCTTGCCGCGCTGGAAGGGAGCACTGATAgg                                           <  1:705527/294‑1 (MQ=255)`  
                                                                                                                                                                                                                                                                    `gATAGTGGTCTTTATGGATGTCATTGTCTATACGAGATG‑CTCCTACGTGAAATCTGAAAGTTAACGGGAGGCATTACGCTAGAATTATTACGTAAGCTAATCCCTTGGGTTCTCGCTGGGATGCTATTCGGGTTAGGATGGCATCTAGGGTCAGACTCAATGGACGCTAAATGGAAACAGGAGGTACACAATGAGTACGTTAAGAGAGTTGAGGCTGCGAAGAGCACTCAAAGAGCAATCGATGCGGTATCTGCTAAGTATCAAGAAGACCTTGCCGCGCTGGAAGGGAGCACTGATAGGa                                          <  1:762474/301‑1 (MQ=255)`  
                                                                                                                                                                                                                                                                    `gATAGTGGTCTTTAGGGATGTCATTGTCTATACGAGATG‑CTCCTACGTGAAATCTGAAAGTTAACGGGAGGCATTACGCTAGAATTTTTACGTAAGCTAATCCCTTGGGTTCTCGCTGGGATGCTATTCGGGTTAGGATGGCATCTAGGGTCAGACTCAATGGACGCTAAATGGAAACAGGAGGTACACAATGAGTACGTTAAGAGAGTTGAGGCTGCGAAGAGCACTCAAAGAGCAATCGATGCGGTATCTGCTAAGTATCAAGAAGACCTTGCCGCGCTGGAAGGGAGCACTGATAGGa                                          <  1:454232/301‑1 (MQ=255)`  
                                                                                                                                                                                                                                                                     `aTAGTGGTCTTTATGGATGTCATTGTCTATACGAGATG‑CTCCTACGTGAAATCTGAAAGTTAACGGGAGGCATTATGCTAGAATTTTTACGTAAGCTAATCCCTTGGGTTCTCGCTGGGATGCTATTCGGGTTAGGATGGCATCTAGGGTCAGACTCAATGGACGCTAAATGGAAACAGGAGGTACACAATTAGTACGTTAAGAGAGTTGAGGCTGCGAAGAGCACTCAAAGAGCAATCGATGCGGTATCTGCTAAGTATCAAGAAGACCTTGCCGCGCTGGAAGGGAGCACTGATAGGat                                         >  1:532382/1‑301 (MQ=255)`  
                                                                                                                                                                                                                                                                     `aTAGTGGTCTTTATGGATGTCATTGTCTATACGAGATG‑CTCCTACGTGAAATCTGAAAGTTAACGGGAGGCATTATGCTAGAATTTTTACGTAAGCTAATCCCTTGGGTTCTCGCTGGGATGCTATTCGGGTTAGGATGGCATCTAGGGTCAGACTCAATGGACGCTAAATGGAAACAGGAGGTACACAATGAGTACGTTAAGAGAGTTGAGGCTGCGAAGAGCACTCAAAGAGCAATCGATGCGGTATCTGCTAAGTATCAAGAAGACCTTGCCGCGCTGGAAGGGAGCACTGATAGGac                                         >  1:433589/1‑300 (MQ=255)`  
                                                                                                                                                                                                                                                                          `ggTCTTTATGGATGTCATTGTCTATACTAGATG‑CTCCTACGTGAAATCTGAAAGTTAACGGGAGGCATTATGCTAGAATTTTTACGTAAGCTAATCCCTTGGGTTCTCGCTGGGATGCTATTCGGGTTAGGATGGCATCTAGGGTCAGACTCAATGGACGCTAAATGGAAACAGGAGGTACACAATGAGTACGTTAAGAGAGTTGAGGCTGCGAAGAGCACTAAAAGAGCAATCGATGCGGTATCTGCTAAGTATCAAGAAGACCTTGCCGCGCTGGAAGGGAGCACTGATAGGATTAttt                                    >  1:143349/1‑301 (MQ=255)`  
                                                                                                                                                                                                                                                                          `ggTCTTTATGGATGTCATTGTCTATACGAGATG‑CTCCTACGTGAAATCTGAAAGTTAACGGGAGGCATTACGCTAGAATTTTTACGTAAGCTAATCCCTTGGGTTCTCGCTGGGATGCTATTCGGGTTAGGATTGCTTCTAGGGTCAGACTCATTGGACGCTAAATGGAACCAGCAGGTACACACTGAGTACGTTAAGAGAGTTGAGGCTGCGAAGAGCACTCAAAGAGTAATCGATGCGGTATCTGCTAAGAATCAAGAAGACCTTGCCGCGCTGGAAGGGAGCAATGATAGTATTAttt                                    >  1:604606/1‑301 (MQ=255)`  
                                                                                                                                                                                                                                                                           `gatgtgtataagagacaggTCTATACGAGATG‑CTCCTACGTGAAATCTGAAAGTTAACGGGAGGCATTATGCTAGAATTTTTACGTAAGCTAATCCCTTGGGTTCTCGCTGGGATGCTATTCGGGTTAGGATGGCATCTAGGGTCAGACTCAATGGACGCTAAATGGAAACAGGAGGTACACAATGAGTACGTTAAGAGAGTTGAGGCTGCGAAGAGCACTCAAAGAGCAATCGATGCGGTATCTGCTAAGTATCAAGAAGACCTTGCCGCGCTGGAAGGGAGCACTGATAGGATTATTTc                                   <  1:665733/283‑1 (MQ=255)`  
                                                                                                                                                                                                                                                                           `gTCTTTATGGATGTCATTGTCTATACGAGATG‑CTCCTACGTGAAATCTGAAAGTTAACGGGAGGCATTATGCTAGAATTTTTACGTAAGCTAATCCCTTGGGTTCTCGCTGGGATGCTATTCGGGTTAGGATGGCATCTAGGGTCAGACTCAATGGACGCTAAATGGAAACAGGAGGTACACAATGAGTACGTTAAGAGAGTTGAGGCTGCGAAGAGCACTCAAAGAGCAATCGATGCGGTATCTGCTAAGTATCAAGAAGACCTTGCCGCGCTGGAAGGGAGCACTGATAGGATTATTTc                                   <  1:563498/301‑1 (MQ=255)`  
                                                                                                                                                                                                                                                                           `gTCTTTATGGATGTCATTGTCTATACGAGATG‑CTCCTACGTGAAATCTGAAAGTTAACGGGAGGCATTATGCTAGAATTTTTACGTAAGCTAATCCCTTGGGTTCTCGCTGGGATGCTATTCGGGTTAGGATGGCATCTAGGGTCAGACTCAATGGACGCTAAATGGAAACAGGAGGTACACAATGAGTACGTTAAGAGAGTTGAGGCTGCGAAGAGCACTAAAAGAGCAATCGATGCGGTATCTGCTAAGTATCAAGAAGACCTTGCCGCGCTGGAAGGGAGCACTGATAGGATTATTTc                                   <  1:554537/301‑1 (MQ=255)`  
                                                                                                                                                                                                                                                                               `ttATGGATGTCATTGTCTATACGAGATG‑CTCCTACGTGAAATCTGAAAGTTAACGGGAGGCATTACGCTAGAATTTTTACGTAAGCTAATCCCTTGGGTTCTCGCTGGGATGCTATTCGGGTTAGGATGGCATCTAGGGTCAGACTCAATGGACGCTAAATGGAAACAGGAGGTACACAATGAGTACGTTAAGAGAGTTGAGGCTGCGAAGAGCACTCAAAGAGCAATCGATGCGGTATCTGCTAAGTATCAAGAAGACCTTGCCGCGCTGGAAGGGAGCACTGATactgtctcttatacc                               >  1:523616/1‑287 (MQ=255)`  
                                                                                                                                                                                                                                                                                `tATTGATGTCATTGTCTATACGAGATG‑CTCCTACGTGAAATCTGAAAGTTAACGGTAGGCATTATGCTAGAATTTTTACGTAAGCTAATCCCTTGGGTTCTCGCTGGGATGCTATTCGGGTTAGGATGGCATCTAGGGTCAGACTCAATGGACGCTAAATGGAAACAGGAGGTACACAATGAGTACGTTAAGAGAGTTGAGGCTGCGAAGAGCACTCAAAGAGCAATCGATGCGGTATCTGCTAAGTATCAAGAAGACCTTGCCGCGCTGGAAGGGAGCACTGATAGGATTATTTCTGAtt                              <  1:284832/301‑1 (MQ=255)`  
                                                                                                                                                                                                                                                                                       `gTCATTGTCTATACGAGATG‑CTCCTACGTGAAATCTGAAAGTTAACGGGAGGCATTATGCTAGAATTTTTACGTAAGCTAATCCCTTGGGTTCTCGCTGGGATGCTATTCGGGTTAGGATGGCATCTAGGGTCAGACTCAATGGACGCTAAATGGAAACAGGAGGTACACAATGAGTACGTTAAGAGAGTTGAGGCTGCGAAGAGCACTCAAAGAGCAATCGATGCGGTATCTGCTAAGTATCAAGAAGACCTTGCCGCGCTGGAAGGGAGCACTGATAGGATTATTTCTGATTTGCGTAg                       >  1:165738/1‑301 (MQ=255)`  
                                                                                                                                                                                                                                                                                          `aTTGTCTATACGAGATG‑CTCCTACGTGAAATCTGAAAGTTAACGGGAGGCATTATGCTAGAATTTTTACGTAAGCTAATCCCTTGGGTTCTCGCTGGGATGCTATTCGGGTTAGGATGGCATCTAGGGTCAGACTCAATGGACGCTAAATGGAAACAGGAGGTACACAATGAGTACGTTAAGAGAGTTGAGGCTGCGAAGAGCACTCAAAGAGCAATCGATGCGGTATCTGCTAAGTATCAAGAAGACCTTGCCGCGCTGGAAGGGAGCACTGATAGGATTATTTCTGATTTGCGTAGCGa                    <  1:514751/301‑1 (MQ=255)`  
                                                                                                                                                                                                                                                                                             `gTCTATACGAGATG‑CTCCTACGTGAAATCTGAAAGTTAACGGGAGGCATTATGCTAGAATTTTTACGTAAGCTAATCCCTTGGGTTCTCGCTGGGATGCTATTCGGGTTAGGATGGCATCTAGGGTCAGACTCAATGGACGCTAAATGGAAACAGGAGGTACACAATGAGTACGTTAAGAGAGTTGAGGCTGCGAAGAGCACTCAAAGAGCAATCGATGCGGTATCTGCTAAGTATCAAGAAGACCTTGCCGCGCTGGAAGGGAGCACTGATAGGATTATTTCTGATTTGCGTAGCGACaa                 >  1:644584/1‑301 (MQ=255)`  
                                                                                                                                                                                                                                                                                             `gTCTATACGAGATG‑CTCCTACGTGAAATCTGAAAGTTAACGGGAGGCATTACGCTAGAATTTTTACGTAAGCTAATCCCTTGGGTTCTCGCTGGGATGCTATTCGGGTTAGGATGGCATCTAGGGTCAGACTCAATGGACGCTAAATGGAAACAGGAGGTACACAATGAGTACGTTAAGAGAGTTGAGGCTGCGAAGAGCACTCAAAGAGCAATCGATGCGGTATCTGCTAAGTATCAAGAAGACCTTGCCGCGCTGGAAGGGAGCACTGATAGGATTATTTCTGATTTGCGTAGCGACaa                 <  1:650627/301‑1 (MQ=255)`  
                                                                                                                                                                                                                                                                                                  `aaCGAGATG‑CTCCTACGTGAAATCTGAAAGTTAACGGGAGGCATTATGCTAGAATTTTTACGTAAGCTAATCCCTTGGGTTCTCGCTGGGATGCTATTCGGGTTAGGATGGCATCTAGGGTCAGACTCAATGGACGCTAAATGGAAACAGGAGGTACACAATGAGTACGTTAAGAGAGTTGAGGCTGCGAAGAGCACTCAAAGAGCAATCGATGCGGTATCTGCTAAGTATCAAGAAGACCTTGCCGCGCTGGAAGGGAGCACTGATAGGATTATTTCTGATTTGCGTAGCGACAATAAGc            <  1:92828/300‑1 (MQ=255)`  
                                                                                                                                                                                                                                                                                                   `aCGAGATG‑CTCCTACGTGAAATCTGAAAGTTAACGGGAGGCATTATGCTAGAATTTTTACGTAAGCTAATCCCTTGGGTTCTCGCTGGGATGCTATTCGGGTTAGGATGGCATCTAGGGTCAGACTCAATGGACGCTAAATGGAAACAGGAGGTACACAATGAGTACGTTAAGAGAGTTGAGGCTGCGAAGAGCACTCAAAGAGCAATCGATGCGGTATCTGCTAAGTATCAAGAAGACCTTGCCGCGCTGGAAGGGAGCACTGATAGGATTATTTCTGATTTGCGTAGCGACAATAAGCg           >  1:689250/1‑301 (MQ=255)`  
                                                                                                                                                                                                                                                                                                   `aCGAGATG‑CTCCTACGTGAAATCTGAAAGTTAACGGGAGGCATTATGCTAGAATTTTTACGTAAGCTAATCCCTTGGGTTCTCGCTGGGATGCTATTCGGGTTAGGATGGCATCTAGGGTCAGACTCAATGGACGCTAAATGGAAACAGGAGGTACACAATGAGTACGTTAAGAGAGTTGAGGCTGCGAAGAGCACTCAAAGAGCAATCGATGCGGTATCTGCTAAGTATCAAGAAGACCTTGCCGCGCTGGAAGGGAGCACTGATAGGATTATTTCTGATTTGCGTAGCGACAATAAGCg           <  1:12725/301‑1 (MQ=255)`  

                                                                                                                                                                                                                                                                                                             `|`                                                                                                                                                                                                                                                                                                              
`CTTAGAGATGTTGGACACTGCGATGGCTCAGCGTATGCTTGCGGACCTTTCGGACCATGAGCGTCGCTCTCCGCAACTCTATAATGCTATTAACAAACTGTTAGACCGCCACAAGTTCCAGATTGGTAAGTTGCAGCCGGATGTTCACATCTTAGGTGGCCTTGCTGGTGCTCTTGAAGAGTACAAAGAGAAAGTCGGTGATAACGGTCTTACGGATGATGATATTTACACATTACAGTGATATACTCAAGGCCACTACAGATAGTGGTCTTTATGGATGTCATTGTCTATACGAGATG‑CTCCTACGTGAAATCTGAAAGTTAACGGGAGGCATTATGCTAGAATTTTTACGTAAGCTAATCCCTTGGGTTCTCGCTGGGATGCTATTCGGGTTAGGATGGCATCTAGGGTCAGACTCAATGGACGCTAAATGGAAACAGGAGGTACACAATGAGTACGTTAAGAGAGTTGAGGCTGCGAAGAGCACTCAAAGAGCAATCGATGCGGTATCTGCTAAGTATCAAGAAGACCTTGCCGCGCTGGAAGGGAGCACTGATAGGATTATTTCTGATTTGCGTAGCGACAATAAGCGGTTGCGCGT  >  NC_001604.1/36582‑37182`

  

Alignment Legend

Aligned base mismatch/match (shaded by quality score): `ATCG/ATCG` `< 3 ≤` `ATCG/ATCG` `< 11 ≤` `ATCG/ATCG` `< 25 ≤` `ATCG/ATCG` `< 36 ≤` `ATCG/ATCG` `< 38 ≤` `ATCG/ATCG`

Unaligned base: `atcg`    Masked matching base: `atcg`    Alignment gap: `‑`    Deleted base: `‑`

Reads not counted as support for junction

read\_name Not counted due to insufficient overlap past the breakpoint.

read\_name Not counted due to not crossing MOB target site duplication.
